# Radial artery pseudoaneurisma due to unintentional arterial puncture during cephalic vein cannulation: A rare case report

**DOI:** 10.1016/j.amsu.2022.103495

**Published:** 2022-03-09

**Authors:** Abdinafic Mohamud Hussein, Abdijlalil Abdullahi Ali, Nazan Bitir, Hassan Kalif Abdi, Ahmed omar Mohamed, Abdirahman Mohamed Hassan Dirie

**Affiliations:** a Department of Cardiovascular Surgery, Mogadishu, Somalia Turkey Recep Tayyip Erdogan Training and Research Hospital, Mogadishu, Somalia; b Department of Pulmonology, Mogadishu, Somalia Turkey Recep Tayyip Erdogan Training and Research Hospital, Mogadishu, Somalia

**Keywords:** Cephalic vein cannulation, Radial artery pseudoaneurysm, Arteriorrhaphy

## Abstract

**Introduction and importance:**

Radial artery pseudoaneurysm is considered as an extremely rare and serious complication that usually follows after cardiac catheterizations with incidence of less than 0.05%, but in lesser frequency with arterial cannulation, trauma, and inflammation or hemodialysis therapy. On the other hand, venous access is clinically important, as it allows for blood sampling, administration of medications, fluids, nutrition, and chemotherapy. But its usage is associated with complications like catheter-associated infections, injuries to peripheral nerves, along with thrombosis and phlebitis of the vessel involved as well as arterial injury.

**Clinical presentation:**

in this report, we present a 67 years old healthy nonsmoker male patient with proximal radial artery pseudoaneurysm following several attempts of cephalic vein cannulation for intravenous access.

**Clinical discussion:**

Radial artery pseudo-aneurisms are very rare with reported incidence of 0.048%, and mainly due to arterial puncture in an attempt of cardiac intervention procedures, but to the authors knowledge this is one of the first reported cases of radial artery pseudoaneurysm caused by arterial puncture in an attempt of cephalic vein cannulation.

**Conclusion:**

Radial artery pseudoaneurysm can occur after attempts of cephalic vein cannulation and patients can be successfully managed with surgical removal of the false aneurism and radial arteriorrhaphy.

## Highlights

• Radial artery pseudoaneurysm can occur in attempt of cephalic vein cannulation.

## 1 Introduction

A pseudoaneurysm is a collection of blood that communicates with the arterial lumen without being enclosed by the arterial wall. With a reported incidence of 0.048%, radial artery pseudoaneurysm is a rare but with serious and disastrous complication, ultimately threatening life and limb. They typically present as expanding pulsatile masses, occasionally causing pain and/or compression of adjacent structures [1]. In this case study, we report a case of radial artery pseudoaneurysm following several attempts of cephalic vein cannulation for intravenous access.

## 2 Case report

A 67 years old healthy male patient presented to our vascular clinic with a complaint of pulsatile mass at the volar aspect of his right forearm just below the antecubital fossa for three months.

The swelling was associated with pain for one month, each of the symptoms were increasing gradually over time. Patient had a history of hypertension for 6 years and was hospitalized 4months prior to his visit due to a transient ischemic attack where he undergone several venous cannulation same site of the affected arm and was taking aspirin afterwards. He denied any history of surgery or trauma at the site and had no history of smoking.

**Fig. 1:**
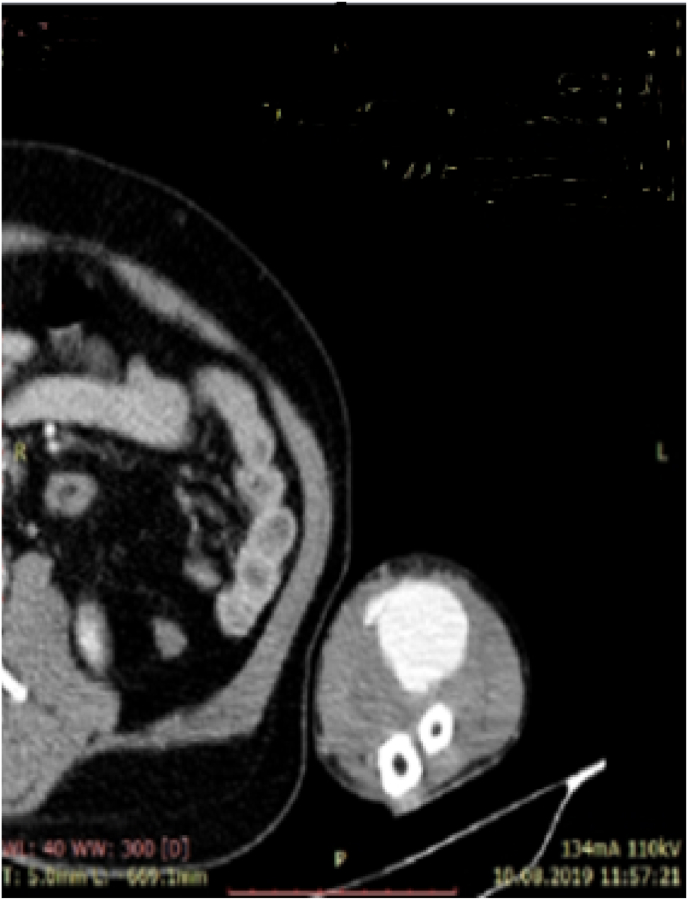
Computed tomography angiography on transverse view Showing Proximal Radial artery pseudoaneurysm.

**Fig. 2:**
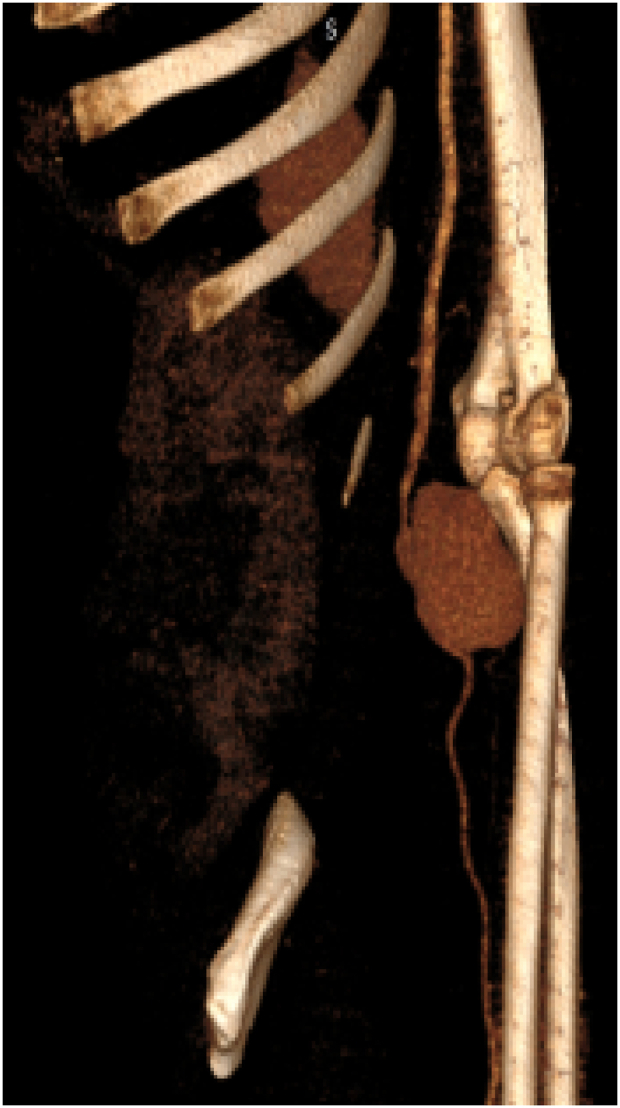
CT-Angiography with 3D-reconstruction image demonstrating Radial artery pseudoaneurysm.

On physical examination, a pulsatile mass of 5.0 by 5.0cm was present at the volar aspect of his right forearm just below the antecubital fossa. Arterial bruit was easily detectible over the mass on auscultation. There were no any scars or skin changes and other arterial examination revealed negative. Allen's test result was bilaterally negative. Performed CT angiography showed the presence of 5 by 5 radial artery pseudoaneurisma just below its origin (see Fig. 2, Fig. 1). Examination of other arteries with Doppler ultrasound showed the absence of un-noticed arterial aneurisms.

**Fig. 3:**
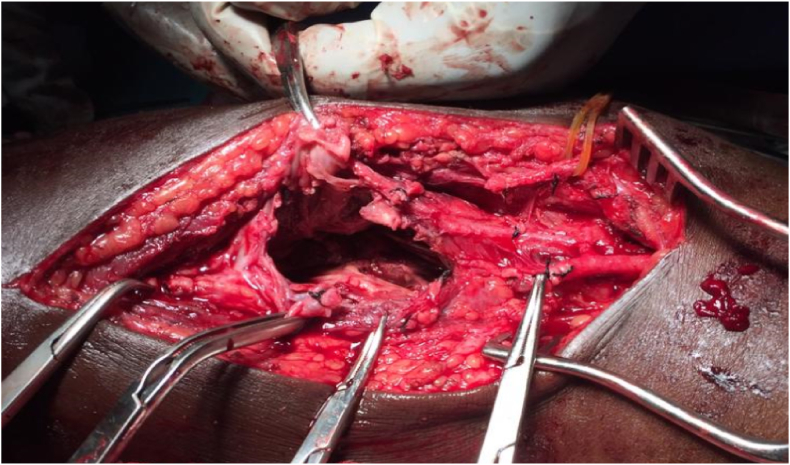
Intraoperative image showing surgical removal of the false aneurism and ligation of the proximal and distal ends of the radial artery.

After discussion with the patient for the pain and possibility of rupture, a decision was made for surgical treatment. The operation was performed by cardiovascular surgery specialist. Under axillary nerve block, an 8 cm longitudinal incision above the aneurysm was done; the pseudoaneurysm was dissected out circumferentially as well as the proximal and distal segments of the radial artery. Each of the proximal and distal segments of the radial artery was controlled using vessel loops. Clamping the proximal aspect of the radial artery, Doppler examination showed positive signals over the thumb and digital arteries indicating sufficient blood follow. Ligation of proximal and distal aspects of the radial artery was followed by aneurism resection (see Fig. 3). Patient was discharged after 2 days of hospital stay without complication and 3 weeks later had resumed to normal daily activities. This case has been reported in line with the SCARE 2020 criteria [2].

## 3 Discussion

An attempt of venous cannulation like cephalic vein can result in radial artery puncture [3]. Main risk factors associated with arterial pseudoaneurysm include use of anticoagulants, antiplatelet agents, age ›65, hypertension, and poor post procedural compression [4]. As reported previously, our patient had hypertension and undergone arterial puncture in attempt of cephalic vein cannulation. He didn't have appropriate compression as well, started using aspirin afterwards. These may be the potential contributing factors of pseudoaneurysm formation in this case.

Although radial artery pseudo-aneurisms are very rare with reported incidence of 0.048%, and mainly due to arterial puncture in an attempt of cardiac intervention procedures, it is also possible to unintentionally puncture radial artery in attempt of cephalic vein cannulation because small number of patients have superficial radial arteries [1,[3], [4], [5]]. To the authors knowledge this is the first reported case of radial artery pseudoaneurysm caused by arterial puncture in an attempt of cephalic vein cannulation.

Conservative treatment has recently become a reliable alternative to surgical intervention in adults with pseudoaneurysm these include ultrasound guided compression repair, reapplication of a compression bandage, percutaneous thrombin injection under ultrasound guidance and clinical observation of the natural course. Surgical intervention is needed in complicated cases (symptomatic, expanding, infection, prolonged history, and with large hematomas), and in patients with failed conservative management. Options include ligation of the artery if distal circulation is not compromised, excision of the pseudoaneurysm, and anastomosis using patch graft [6]. Due to the painful, prolonged and large pseudoaneurysm, we operated our patient with excision of pseudoaneurysm and ligature of the radial artery after we checked the patency of the palmer collateral circle by Allen's test and with a handheld Doppler ultrasound. Follow up at 1 months showed no symptoms with patency of the radial artery.

## 4 Conclusion

From this study, we conclude that radial artery pseudoaneurysm can occur after attempts of cephalic vein cannulation. Patient can be successfully managed with surgical removal of the false aneurism and radial arteriorrhaphy.

## Ethical approval

N/A.

## Funding source

There is no funding source for this study.

## Author contribution

All authors contributed toward writing, analysis, drafting, and revising of the paper.

## Conflicts of interest

The authors declare that there is no competing interest related to the study, authors, other individuals, or organizations.

## Consent to participate

Written informed consent was obtained from the patient for publication of this case report and accompanying images. A copy of the written consent is available for review by the Editor-in-Chief of this journal on request.

## Provenance and peer review

Not commissioned, externally peer reviewed.

## Guarantor

Abdinafic Mohamud Hussein.

## Ethical approval

According to our hospital rule, Ethical approval is only required in articles but not case reports.

## Sources of funding for your research

There is no funding source for this study.

## Author contribution

All authors contributed toward writing, analysis, drafting, and revising the paper and they gave final approval of the version to be published, and agree to be accountable for all aspects of the work.

## Consent

Written informed consent was obtained from the patient for publication of this case report and accompanying images. A copy of the written consent is available for review by the Editor-in-Chief of this journal on request.

## Registration of research studies

1. Name of the registry: Not applicable
2. Unique Identifying number or registration ID: Not applicable
3. Hyperlink to your specific registration (must be publicly accessible and will be checked): Not applicable

## Guarantor

Abdinafic Mohamud Hussein.

## Declaration of competing interest

The authors declare that there is no competing interest related to the study, authors, other individuals or organizations.
